# Long-term survival after total pelvic exenteration in a patient with recurrent cervical carcinoma: A case report

**DOI:** 10.4274/tjod.04207

**Published:** 2014-09-15

**Authors:** Soner Düzgüner, Tuba Zengin, Tolga Taşçı, Taner Turan, Nurettin Boran, Gökhan Tulunay, Mehmet Faruk Köse

**Affiliations:** 1 Dr. Sami Ulus Women’s Health Teaching and Research Hospital, Clinic of Obstetrics and Gynecology, Ankara, Turkey; 2 Etlik Zübeyde Hanım Women’s Health Teaching and Research Hospital, Clinic of Gynecologic and Oncology, Ankara, Turkey; 3 Bahçeşehir University Faculty of Medicine, Department of Obstetrics and Gynecology, İstanbul, Turkey

**Keywords:** Cervical adenocarcinoma, exenteration, recurrence

## Abstract

The management of recurrent cervical cancer depends mainly on previous treatment as well as on the site and extent of recurrence. Pelvic exenteration usually represents the only therapeutic approach with curative intent for women with central pelvic relapse who have previously received irradiation. In the present report, we share our experience regarding survival outcome in a patient with recurrent endocervical carcinoma who underwent total pelvic exenteration.

## INTRODUCTION

Cervical malignancy is the second most common malignancy in women worldwide^(1)^. Up to 25% of women with FIGO stage IB-IIA cervical cancer may recur after initial therapy^(2)^. Frequently, these recurrences may be treated with radiotherapy; however, radical surgery may offer an alternative for curative treatment. Long-term survival is directly correlated with complete tumor resection, so establishing resectability is a key aspect of preoperative planning^(3)^.

Pelvic exenteration (PE) is an ultraradical surgery that was first described by Brunschwig in 1948^(4)^. This surgery involves en bloc resection of the pelvic organs, including the internal reproductive organs, bladder, and rectosigmoid. Traditionally, PE has been used for centrally recurrent cervical carcinoma^(5)^.

The purpose of the present case is to share our experience of total pelvic exenteration (TPE) in a patient with recurrent endocervical carcinoma regarding survival outcome.

## CASE

A 51-year-old woman underwent type 1 hysterectomy at outer center, has been reported with histopathology of leiomyoma uteri and endocervical adenocarcinoma. She has been observed without treatment for 15 months until presenting with abnormal vaginal bleeding at outer center. Vaginal cuff biopsy has revealed adenocarcinoma. Due to recurrence of cervical carcinoma, she has undergone pelvic radiotherapy and vaginal brachytherapy with 7 courses of weekly cisplatin (concurrent chemoradiotherapy). After 6 months of following chemoradiotherapy, on account of recurrent complaint of vaginal bleeding and cuff biopsy with histopathology of adenocarcinoma, patient has been referred to our clinic for advanced evaluation and management.

Pelvic examination revealed fullness (size 35x20 mm) on vaginal cuff and infiltration of left parametrium. Irregularity and more wall thickening at the right superolateral side of the vaginal cuff, nearly 23x22 mm, were detected on magnetic resonance imaging (MRI). MRI of the upper abdomen and computerized tomography (CT) of thorax confirmed no residual mass.

**Figure 1:**
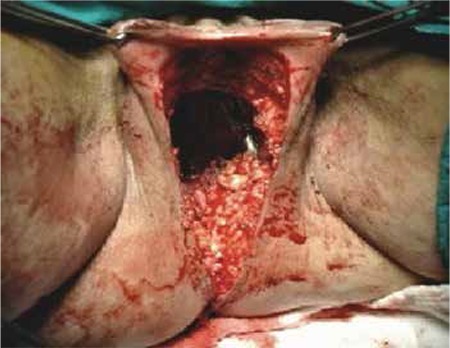
Exenteration; Perineal region

Based on the findings and clinical status of the patient, we decided to perform surgery of exenteration as a therapeutic option. We performed TPE + paraaortic lymphadenectomy + indiana pouch + ileocolic anastomosis + colostomy + omental J-plasty (Figure 1). Histopathology of the paraffin section of specimens was reported as metastasis of adenocarcinoma (grade 1) to the vagina, the left ovary and right tuba uterine. The surgical border on the lower margin of the vagina was free. We did not detect any short or long-term morbidity due to surgery.

Adjuvant chemotherapy with paclitaxel and cisplatin was given due to ovarian metastasis. After premedication, initially paclitaxel (175 mg/m^2^) was infused in 3 hours. Then, cisplatin (75 mg/m^2^) was infused in 2 hours. Six courses of this combination were given every 3 weeks. The complete clinical response was obtained after treatment.

Patient was evaluated every 3 months for the first 2 years, every 6 months for the following 3 years and annually thereafter. Follow-up included physical examination, abdominal sonography (abdominal MRI at first year and then if necessary), chest X-ray (every year or if necessary), complete blood count, serum biochemistry and CA-125 level (because of ovarian metastasis). There was no recurrence of the disease in follow-up. The patient is alive with no evidence of recurrence for more than 80 months after TPE.

## DISCUSSION

The management of recurrent cervical cancer depends mainly on previous treatment and on the site and extent of recurrence^(6,7)^. In the present case, the patient has recently completed a treatment of chemoradiation after central pelvic recurrence, around vaginal cuff, has been determined 15 months after type 1 hysterectomy.

The original classification of PE into three groups, i.e., anterior (removal of the bladder and internal reproductive organs but spares the gastrointestinal tract), posterior (remove the internal reproductive organs and the rectosigmoid but spares the anterior vagina, urinary bladder, and ureters) and total (anterior + posterior), addresses only the nature of the pelvic viscera removed. The patient underwent total pelvic exenteration in our case.

In the literature, there are specific criteria for surgical resection. Patients with resectable central recurrences that involve the bladder and/or rectum, without evidence of intraperitoneal or extra-pelvic spread, and who have a dissectible tumor-free space along the pelvic side wall are potentially suitable for exenteration^(7)^. Similarly, one study defined the candidates for exenteration is those with central local recurrences that have not extended to the pelvic sidewalls^(6)^. In addition, in the same study, it was noticed that all patients who have undergone previous radiation therapy should be considered for surgical resection for centrally located recurrences. According to Shingleton et al., the best candidates for cure by PE were the patients with recurrent small (<3 cm), mobile central tumors and with a disease free interval of one year or longer^(8)^. In the present case, our indicated parameters for TPE were no documented distant metastasis, central pelvic recurrence with no extension to the pelvic sidewall, tumor size smaller than 3 cm and the history of previous radiation therapy.

PE is a major surgery with significant morbidity^(5)^. The clinical improvements obtained in the last decades may be mainly due to better surgical techniques and more intensive postoperative care. We have no detected any complication or morbidity after operation. One study noticed that the more strict criteria for the patient selection (central disease, no paraaortic involvement, no peritoneal disease), the greater the chance for a favorable clinical outcome^(9)^. A better definition of patient selection criteria made easier by the availability of new diagnostic techniques such as positron emission tomography scanning (PET scan), CT, MRI^(10,11)^. The patient was pre-operatively evaluated by MRI of the upper and lower abdomen, and CT of thorax for metastasis and recurrence.

PE remains the only therapeutic option that offers the possibility of long-term survival for most patients^(6)^. In a recent series, the 5-year overall survival after PE ranged from 22% to 48%, respectively^(3,8,12,13)^. In presented case, patient is alive and free of disease for more than 80 months after TPE. Free surgical margins^(3,8,12)^, negative lymph nodes^(12,14)^, small tumor size^(8)^ and long disease-free interval^(3,8,12)^ were associated with a more favorable prognosis. In our case, histopathological findings after PE surgery have been reported as negative lymph nodes, level of lower vagina margins remained free, metastasis to the left ovary and right tuba uterina. Independent from stage of the disease ovarian metastasis is a poor prognostic factor in cervical carcinoma^(15)^. It leads to a regression of overall survival with decrease in quality of life. Despite the presence of ovarian metastasis, the patient lives for more than six years with no evidence of disease recurrence.

Marnitz et al. reported that survival correlated significantly with the time interval between primary treatment and recurrence^(3)^. In that study, 5-year survival was 16.8% for disease failure in first 2 years, 28% for 2-5 years and 83.2% for after 5 years. In the present case, recurrence after primary surgery was identified 15 months later. According to the study of Mourton et al., 68% of patients subsequently developed recurrence after TPE. The median time from TPE to recurrence was 7 months (range 2-73 months), 92% occurring within 2 years^(16)^. In the presented case, no evidence with recurrence was noticed for 80 months after exenteration. One study showed that affecting factors for overall survival were resection margin status, pelvic wall and rectal involvement^(17)^. In our case, there were no pelvic wall and rectal involvement.

At present, pelvic reconstructive procedures are strongly recommended after exenteration. An ileocolonic segment is currently employed for continent urinary diversion^(18)^. Indiana pouch (Indiana continent urinary reservoir), firstly described by Rowland et al. in 1987^(19)^, was performed to our case. Colostomy was performed for fecal diversion. Omental J-plasty was performed for pelvic floor coverage.

In conclusion, the present case showed that total pelvic exenteration has a potential to provide long-term survival despite the second recurrence which is seen in 2 years after primary surgery and presence of ovarian metastasis.
